# Predictive Modeling of Ungulate–Vehicle Collision in the Republic of Korea

**DOI:** 10.3390/biology12081068

**Published:** 2023-07-30

**Authors:** Kyungmin Kim, Desiree Andersen, Yikweon Jang

**Affiliations:** 1 Interdisciplinary Program of EcoCreative, Ewha Womans University, Seoul 03760, Republic of Korea; kkim193@ewhain.net; 2 Department of Life Sciences and Division of EcoScience, Ewha Womans University, Seoul 03760, Republic of Korea

**Keywords:** ungulate–vehicle collision, roadkill, fragmentation, spatial modeling, seasonal behavior

## Abstract

**Simple Summary:**

Ungulate–vehicle collisions (UVC) often threaten human life and property due to the large body size of ungulates. The purpose of this study is to understand factors contributing to UVC of three ungulate species (*Capreolus pygargus*, *Hydropotes inermis*, and *Sus scrofa*) in the Republic of Korea using predictive modeling. We relied on the UVC data of the Korea Roadkill Observation System, a government-sponsored web-based system that is required for road workers to report roadkill incidents on all major road types across the country with a standardized data collection method. There were 25,755 UVC datapoints between 2019 and 2021. The UVC frequencies tended to be the highest in the most active seasons of the year, such as dispersal or mating seasons. Factors critical for UVC frequencies were different among three ungulate species, suggesting individualized mitigation plans.

**Abstract:**

Animal–vehicle collisions (AVC) threaten animals as well as human life and property. AVC with ungulates, called ungulate–vehicle collision (UVC), often seriously endangers human safety because of the considerable body size of ungulates. In the Republic of Korea, three ungulate species, *Capreolus pygargus*, *Hydropotes inermis*, and *Sus scrofa*, account for a large proportion of AVC. This study aimed to understand the characteristics of UVC by examining various parameters related to habitat, traffic, and seasonality using MaxEnt. The results showed that the peak UVC seasons coincided with the most active seasonal behaviors of the studied ungulates. For the modeling results, in *C. pygargus*, habitat variables are most important for models across seasons, and UVC events are most likely to occur in high mountain chains. In *H. inermis*, habitat and traffic variables are most important for models across seasons. Although the important habitat for the models were different across seasons for *S. scrofa*, the maximum speed was consistently critical for models across all seasons. Factors critical to UVC in the Republic of Korea were different for the three ungulate species and across seasons, indicating that seasonal behavior should be considered along with landscape and traffic characteristics to mitigate UVC.

## 1. Introduction

The expansion of linear infrastructure causes habitat fragmentation and reduces habitat connectivity. The consequent increase in collisions between vehicles and wildlife, known as animal–vehicle collision (AVC), negatively impacts human and wildlife safety. The risk to human safety increases with the body size of the wildlife involved in AVC [1,2]. The body size of ungulates is typically larger than that of other species involved in AVC [3]. Thus, ungulate–vehicle collision (UVC) is a globally important issue [4,5]. The dangers of UVC have been demonstrated in previous studies. Among the reported traffic accidents involving UVC in the United States and Canada, 2.8–9.7% resulted in human injury [6]. In Brazil, AVC with large animals, such as deer and horses, accounts for 3.3% of total vehicle accidents, and 18.5% of these accidents lead to human injury or fatality [7]. In addition, 300 human fatalities and 30,000 injuries involving UVC occur annually in Europe [8]. Thus, understanding the spatial and temporal characteristics of UVC is important for reducing the UVC frequency for wildlife conservation as well as for human safety.

There are many variables that are related to AVC, such as population density, landscape, traffic, and seasonality. Among them, except population density, data of the three variables are often available and considered critical [9]. The distribution of an animal population is often clumped, depending on resource availability, which is dictated by landscape features. Animals often require different types of resources for reproduction and survival. For example, animals often rest in a patch that may be hidden away from threats and feed on a different patch for food [10,11]. Disconnectedness among patches of resources forces animals to move regularly. Accordingly, landscape features are often the most important factors predicting UVC [12,13]. The best model predicting the UVC of European roe deer (*Capreolus capreolus*) in France included landscape covariates and habitat connectivity [14]. In addition, the UVC of *Odocoileus* spp. is more likely to occur close to water and non-forested vegetation in the United States of America (USA), possibly because of habitat preference [15].

Traffic also plays an important role in predicting the occurrence of AVC. Depending on the nature of the use, each road has distinctive characteristics. In general, major roads connecting large cities have many lanes with high traffic volumes, whereas roads located in the countryside around villages have fewer lanes with low traffic volumes and low maximum speeds. When an animal attempts to cross a road, its likelihood of passing the road successfully depends on various conditions, such as traffic volume, road structure, and the number of lanes on the road, and their impact varies by species. For instance, during the lockdown due to the COVID-19 pandemic, significantly fewer UVC of roe deer and wild boar were observed due to lower traffic volume in Slovenia [16]. In contrast, traffic had no impact on the UVC of the mule deer *Odocoileus hemionus* in the USA [17]. In the Republic of Korea, most cases of UVC of the water deer *Hydropotes inermis* were located close to road connections, such as ramps and interchanges [18], indicating a high risk of UVC around areas where the species enters the road. 

Animal movements are often seasonal, which is an important factor in AVC [18,19,20]. Animals are often most active during their breeding season. In general, mammalian males expand their home range and/or make long-distance movements to find mates at the beginning of the breeding season [21,22,23], making males vulnerable to AVC [19,24]. In addition, juveniles often disperse from their natal home range to reduce competitions with adult individuals [25]. Inexperienced juveniles often lack knowledge of high-traffic roads, which results in AVC [9]. Therefore, animal mortality due to AVC fluctuates seasonally. For instance, the number of *H. inermis* UVC is significantly different across months and seasons according to their seasonal behaviors [18]. In addition, *Odocoileus hemionus* UVC varied significantly by season and month because of its high activity during the mating season and reduced movement during spring owing to high food availability [17].

Five species of ungulates are native to the Republic of Korea: the Siberian roe deer *Capreolus pygargus*, water deer *H. inermis*, wild boar *Sus scrofa*, musk deer *Moschus moschiferus*, and long-tailed goral *Naemorhedus caudatus* [26]. Among them, *M. moschiferus* and *N. caudatus* are very rare and only regionally distributed; therefore, they are designated as endangered species. The other three species, *C. pygargus*, *H. inermis*, and *S. scrofa*, are comparably abundant throughout the country. Accordingly, these three species are the focus of this study.

*Capreolus pygargus* is distributed from the Republic of Korea mainland to southern Jeju Island. However, the mainland and island populations have different morphological characteristics and population densities. The mainland population weighs between 28 and 35 kg and has a population density of 1.9 individuals per km^2^, according to a long-term national mammal monitoring program run by the Korean government [26]. Meanwhile, the population in Jeju is smaller than that in the mainland population, weighs an average of 17 kg, and has a population density of 5.3 individuals per km^2^ [26,27]. On the mainland, *C. pygargus* inhabits forest interiors with elevations between 400 and 600 m [28]. Fawns are born between May and July, and the rutting season starts in August. During the breeding season, males compete with other males to defend their territories. In doing so, they often approach the edges of their home ranges, which are typically bordered by roads and streams [26,29].

*Hydropotes inermis*, weighing 16–21 kg, is the smallest ungulate species in the country and is found only on the mainland [26]. It is an edge species, inhabiting various types of land cover, including forests, rice fields, and wetlands [26]. Although this species is listed as vulnerable by the IUCN Red List, it is the most abundant wildlife and its population is rapidly increasing in the Republic of Korea [30,31]. The population density of *H. inermis* is 7.7 individuals per km^2^ on average, with the highest densities observed in the lowlands. Thus, it is reasonable that *H. inermis* is the most common UVC victim in the country [32]. Regarding seasonal behavior, two to six *H. inermis* fawns are born in May and June in the temperate region. Yearlings spend one year with their mother and then disperse to their own territory between May and June [26,33].

*Sus scrofa* is one of the largest terrestrial mammals in the Republic of Korea, with weights ranging from 45 to 300 kg [26]. The species was considered extinct in Jeju Island in the past, but many individuals, that are presumed to be escaped from farmland, have been observed recently [34]. *Sus scrofa* mainly inhabits the forest interiors. This species has recently become numerous, from 1.3 individuals per km^2^ in 1978 to 3.7 individuals per km^2^ in 2021 [31]. Accordingly, they are often found in urban environments [35]. Therefore, this species has been designated as a culling species because of constant conflicts with humans. The mating season for *S. scrofa* peaks between November and December. The species is omnivorous, mainly consuming vegetable matter, some invertebrates, snakes, and occasionally large vertebrates.

Landscape features that may be important for species occurrence are heterogeneous. Thus, it is difficult to generalize the findings from the AVC datasets that are collected on regional scales. Thus, AVC datasets that are broad enough, exceeding the ranges of species distribution, are ideal to provide information about the likelihood of AVC at a large spatial scale. However, such nationwide datasets are difficult to obtain because of high cost and the difficulty of organizing collection efforts. Many AVC studies rely on police reports, due to availability, but such police data is produced only when an accident is reported to the police. Accordingly, an unknown proportion of AVC incidents may go unreported. Citizen science is another venue to collect AVC data at a larger temporal and spatial scale. However, data from a citizen science program may be opportunistic and may be biased, depending on levels of participant training. To overcome the difficulties of collecting AVC data, a standardized protocol with a great deal of surveyors at a large scale may be helpful.

In the Republic of Korea, the Korea Roadkill Observation System (KROS; nie-ecobank.kr/) was launched by the government to integrate and manage scattered AVC data [32]. KROS covers all road types across the country with a standardized data collection method. In addition, it is mandatory for road workers of all road authorities in the country to use KROS whenever they find animal carcasses on roads. Thus, the KROS dataset is considered to be one of the largest, with high data quality that can be useful to AVC mitigation policy in the country.

Habitat modeling techniques have been incorporated to predict the occurrence of AVC. MaxEnt (maximum entropy [36]) is a machine learning algorithm that predicts habitat suitability based on the response of presence points to environmental conditions relative to the response of background points. Thus, the MaxEnt modeling is used to predict the expected presence as an index of suitability. Because of its predictive power, MaxEnt can also be used to predict the probability of occurrences besides species presence (e.g., in physics [37]; socioeconomics [38]; and groundwater mapping [39]) using spatial data and appropriate predictor variables. For this reason, some recent studies have utilized MaxEnt to predict AVC risk and have concluded that it is useful in identifying important variables influencing AVC [40,41,42,43,44,45,46].

The aim of this study is to understand UVC characteristics by evaluating variables related to the surrounding landscape, traffic, and animal seasonality, using one of the largest and most reliable country-wide AVC datasets in the world. To this end, we developed predictive seasonal models of UVC for three species across a road network in the Republic of Korea using MaxEnt. Based on our results, we identified the critical factors influencing UVC, which will be useful in reducing the threat to human safety.

## 2. Materials and Methods

### 2.1. Study Area 

**Figure 1:**
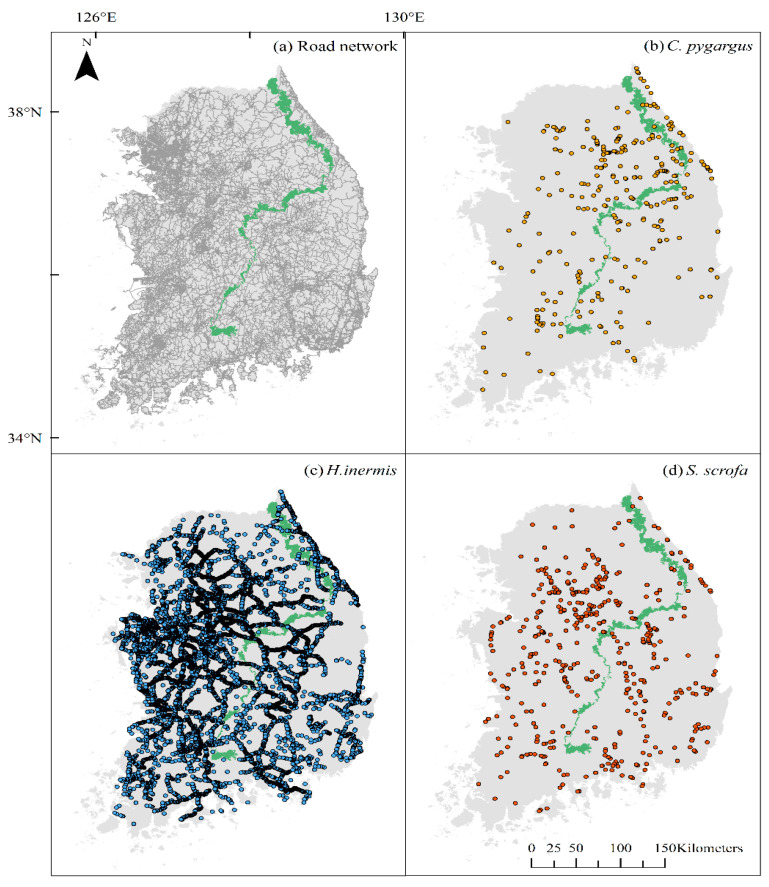
The Republic of Korea mainland with the road networks of all seven types. (**a**) The Baekdu-daegan mountain chain, which is a series of high mountains, is shown in green. Clusters of roads in gray are major cities. The UVC locations of *Capreolus pygargus* (**b**), *Hydropotes inermis* (**c**), and *Sus scrofa* (**d**) between 2019 and 2021 are shown using dots on the country map. The maps were generated using ArcGIS Desktop 10.8.1.

In the Republic of Korea, according to the Korean Statistical Information Service, there are 105,083 km of roads as of 2021 (Figure 1). There are seven types of roads in Korea: expressways, national highways, metropolitan roads, provincial roads, municipal roads, county roads, and district roads. The expressway and national highway are inter-provincial roads, whereas the rest extend only within their respective administrative levels. An expressway is a toll road with a speed limit above 100 km/h and is managed by the Korea Expressway Corporation. A national highway connects major cities with a speed limit of up to 80 km/h and is maintained by the Ministry of Land, Infrastructure and Transport (MOLIT), Korea. A metropolitan road is located in a metropolitan city, and a provincial road is an arterial road in a province. Municipal, county, and district roads are typically small-scale roads with low speed limits and are located inside the municipality, county, and district, respectively.

Topographically, the Republic of Korea is dominated by the Baekdu-daegan mountain chain, which has numerous peaks over 1000 m. The mountain chain runs along the east coast and turns into the midland of the peninsula to the south. Because of this mountain chain, rivers generally flow westwards or southwards; therefore, alluvial plains have developed in the west and south. Large cities with dense networks of roads are well-developed in these plains. (Figure 1). In this study, we only focused on the mainland and excluded Jeju Island, which is located in the most Southern part of the country, due to heterogeneity in environmental conditions such as climate, geographical features, and vegetation that may influence the results.

### 2.2. Data Collection

A smartphone application linked with KROS was developed and released for use by more than 5000 road menders and related managers affiliated with road authorities who were in charge of removing animal carcasses from the roads. Road menders took pictures whenever a carcass was found while patrolling the roads or when a civil complaint about AVC was received. Along with pictures, location and time information were conveyed to the KROS website in real time. Then, wildlife experts identified the species and correct location of each data point based on the picture for data cleaning. The UVC data for *C. pygargus*, *H. inermis*, and *S. scrofa* used in this study were derived from the 2019–2021 KROS data (Figure 1). Road data required for spatial analyses were obtained from the National Transport Information Center.

### 2.3. Habitat Suitability Modeling

To map the probabilities of UVC for three species of ungulates (*C. pygargus*, *H. inermis*, *S. scrofa*), habitat suitability models (HSMs) were created for all species. For habitat suitability modeling, we applied the MaxEnt software, which uses presence data to compute habitat suitability using machine learning. We used occurrence point data obtained from the Global Biodiversity Information Facility [47] (GBIF), with a bounding area of the Republic of Korea. After removing occurrences with coordinate uncertainty greater than the resolution of our environmental layers (0.001 decimal degrees), we retained 5178 occurrences of *C. pygargus*, 35,050 occurrences of *H. inermis*, and 13,088 occurrences of *S. scrofa* for modeling. The HSMs were trained using environmental layers (Table 1) with a resolution of 0.001 decimal degrees, including bioclimatic variables (Worldclim.org ver. 1.4) [48] and habitat-related variables (land cover type (Table 2), distance to forest, distance to water, distance to urban areas, elevation, slope, ruggedness, distance to streams and coast, tasseled-cap greenness, and wetness). Duplicate presence records were removed as an option in the modeling software to reduce the spatial autocorrelation caused by sampling bias. The models were trained with five replicates using the cross-validate run type, with 80% of the points used for training and 20% used for testing. The average of the five replicates was used as the final model. Variables were reduced based on percent contribution (>5; contribution in building the model) or permutation importance (>5; post-hoc calculated contribution to final model) within the initial full model. Final HSMs were validated using the area under the receiver operating curve (AUC) and true scale statistics (TSS) [49].

### 2.4. UVC Mapping

MaxEnt was used to model the UVC probabilities of the three ungulate species in the Republic of Korea. Environmental layers included those used to build the HSMs (Table 1; excluding bioclimatic variables except annual mean temperature and annual precipitation), final HSMs, traffic-related variables (number of lanes and maximum speed), and Euclidean distance to wildlife crossings. These layers were masked (extracted) using road layers, because UVC can only occur along roads. Therefore, background points generated by MaxEnt falling elsewhere would not represent a true lack of UVC along the roads. As with the HSMs above, five replicates using the cross-validation run type were averaged for the final maps of the UVC probability. However, when we tested correlation using spearman’s rho, HSM of *S*. *scrofa* was excluded because of its high correlation with other environmental variables with higher contributions to the models (r > |0.75|). Removal of this variable served to reduce the potential compounded uncertainty arising from models including such highly correlated variables. To understand how seasonal changes influence UVC probability, models of UVC probability for each species were created with data separated by season (spring: March–May; summer: June–August; fall: September–November; winter: December–February). Duplicate presence points were not removed (MaxEnt setting to remove non-independent occurrence points occupying the same cell of the environmental data, reducing spatial sampling bias) because UVC occurrences could be considered independent observations (i.e., not multiple counts of the same individual). Finally, we set the default prevalence of each group (by species and season) to be proportional to their respective prevalence (percentage) within the whole dataset. The final models were assessed using AUC and TSS. Spatial analysis was conducted using ArcGIS Desktop 10.8.1 and ArcGIS Pro 2.8.0.

## 3. Results

### 3.1. UVC Data

**Figure 2:**
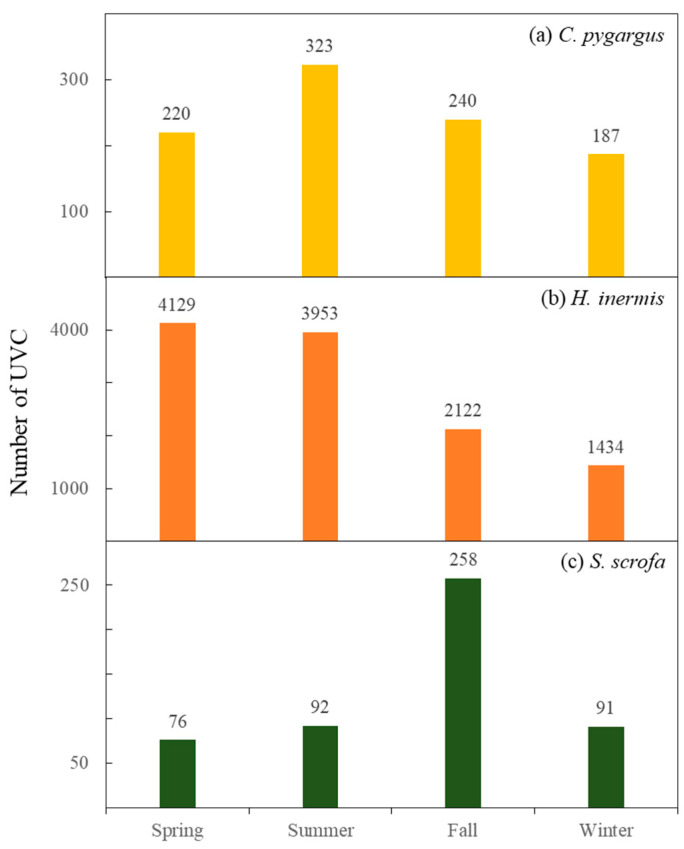
The number of georeferenced ungulate–vehicle collision (UVC) events of (**a**) *C. pygargus*, (**b**) *H*. *inermis*, and (**c**) *S. scrofa* by season between 2019 and 2021 in the Republic of Korea (collected from KROS). Spring: March–May; Summer: June–August; Fall: September–November; Winter: December–February.

Between 2019 and 2021, there were 970 *C. pygargus* (mainland only), 24,268 *H. inermis*, and 517 *S. scrofa* datapoints in the georeferenced UVC dataset collected through KROS. In general, the UVC frequencies of each species were seasonal (Figure 2). The UVC frequency of *C. pygargus* was the highest in summer, which is the rutting season of the species, and was 1.7 times higher in summer than that in the lowest season—winter. The UVC frequency of *H. inermis* peaked in spring, which is the dispersal season of juveniles, and was the lowest in winter, with a difference of 2.9 times. For *S. scrofa*, the UVC frequency was the highest in fall, which is the beginning of the rutting period as well as the high-activity season to prepare for overwintering, and the lowest in spring, with a difference of 3.4 times.

### 3.2. Habitat Suitability Models

The habitat suitability models showed a good fit for *C. pygargus* (average AUC values of 0.802 ± 0.007) and *S. scrofa* (0.782 ± 0.012) but a poor fit for *H. inermis* (0.602 ± 0.004). However, this model was generally acceptable for *H. inermis*, which is a habitat generalist. For *C. pygargus*, elevation had the highest contribution, followed by the maximum temperature of the warmest month, and the minimum temperature of the coldest month had high permutation importance. For *H*. *inermis*, the mean temperature of the wettest quarter had the highest contribution, followed by ruggedness, and the slope had a high permutation importance. For *S*. *scrofa*, elevation had the highest contribution, followed by land cover and precipitation of the coldest quarter, and greenness had high permutation importance. In short, important variables for the habitat model of *C. pygargus* were mainly related to climate, those of *H. inermis* were related to climate and topography, and those of *S. scrofa* were highly variable.

### 3.3. UVC Probabilities

**Figure 3:**
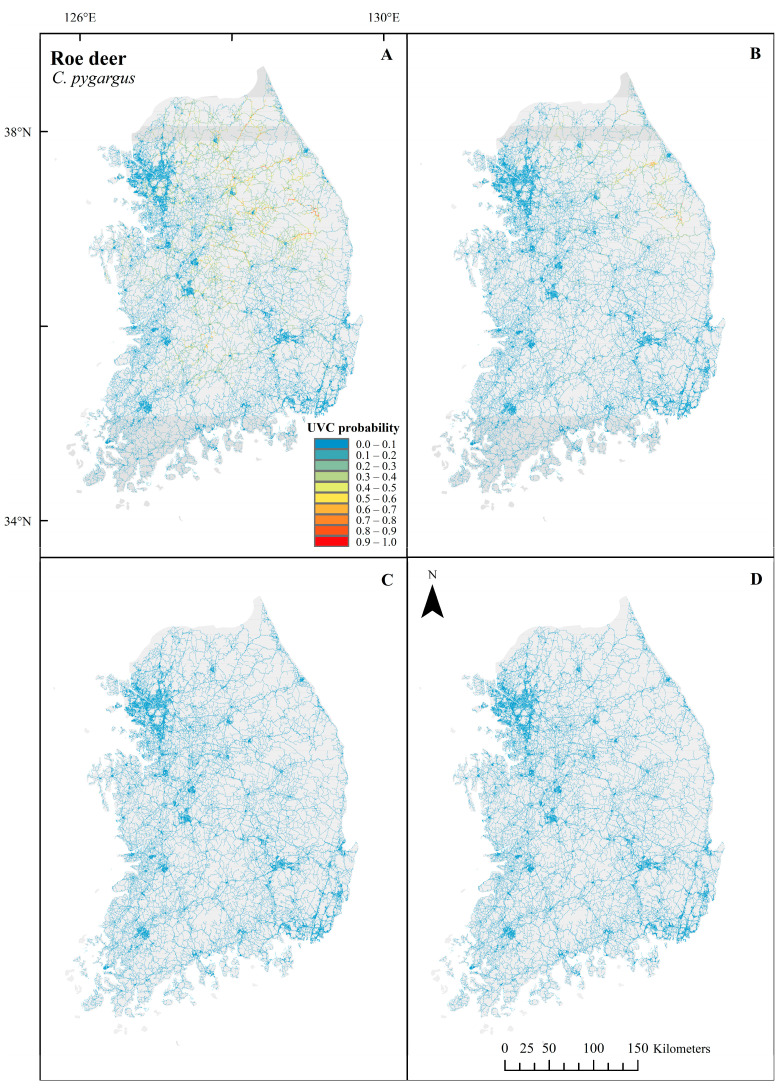
Season-based UVC probability predictions of *C. pygargus* in spring (**A**), summer (**B**), fall (**C**) and winter (**D**) along roads using UVC data between 2019 and 2021 in the Republic of Korea. Red and blue colors indicate a high and a low probability of UVC, respectively. The maps were generated using ArcGIS Desktop 10.8.1.

**Figure 4:**
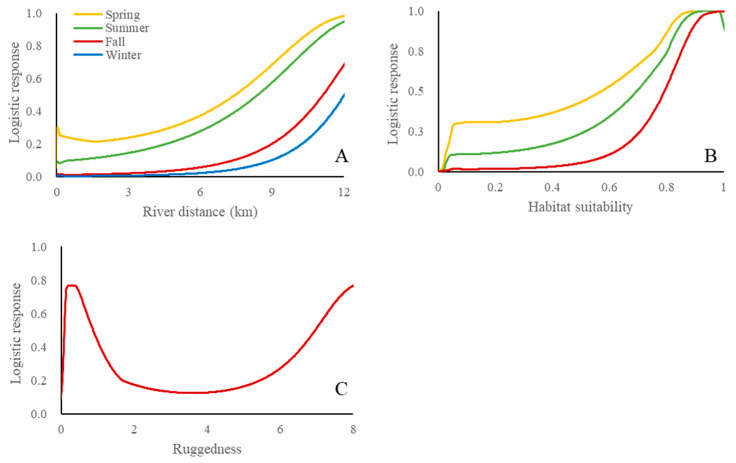
Response curves of river distance (**A**), habitat suitability (**B**) and ruggedness (**C**) in each season for *C. pygargus* using UVC data between 2019 and 2021 in the Republic of Korea. Each response curve of predicted probability is independent of other variables in the models.

The predictive powers of the models evaluated using AUC values were considered acceptable (>0.75) for all 12 models (Table 3). In general, all UVC maps of the three species showed a common pattern of low probability in urbanized regions throughout the country. In *C. pygargus*, UVC probabilities were high around the Baekdu-daegan mountain chain in spring and summer, whereas they were low in fall and winter throughout the country (Figure 3). Important variables in the UVC predictive models of *C. pygargus* were highly related to habitats but were not related to traffic across seasons (Figure 4 and Table 4). In the spring and summer models, the variables with high permutation importance (>10) were habitat suitability and river distance. As habitat suitability and distance to the river increased, the likelihood of UVC also increased monotonically. In the fall model, habitat suitability had the highest permutation importance, followed by river distance and ruggedness. The likelihood of UVC probability was bimodal, with peaks of very low and high ruggedness. For winter, only river distance showed high permutation importance. In *C. pygargus*, habitat-related variables were the most important for UVC models across seasons, and UVC events were most likely to occur in the high mountain chain.

**Figure 5:**
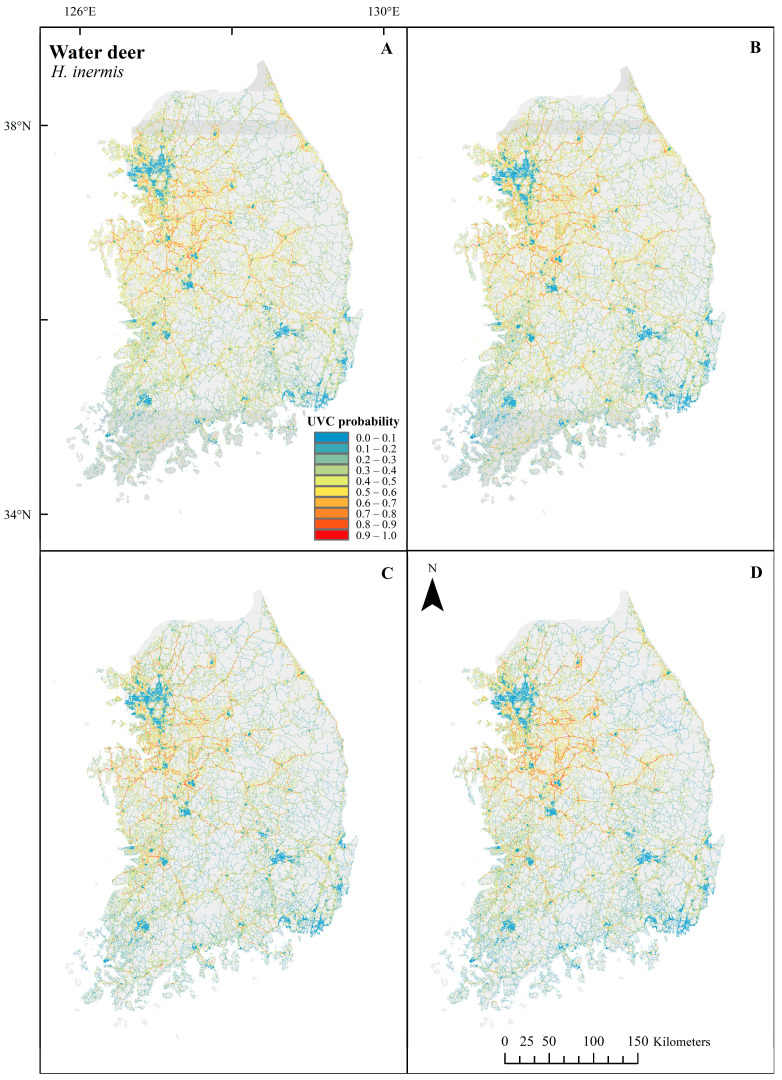
Season-based UVC probability predictions of *H. inermis* in spring (**A**), summer (**B**), fall (**C**) and winter (**D**) along roads using UVC data between 2019 and 2021 in the Republic of Korea. Red and blue colors indicate a high and a low probability of UVC, respectively. The maps were generated using ArcGIS Desktop 10.8.1.

**Figure 6:**
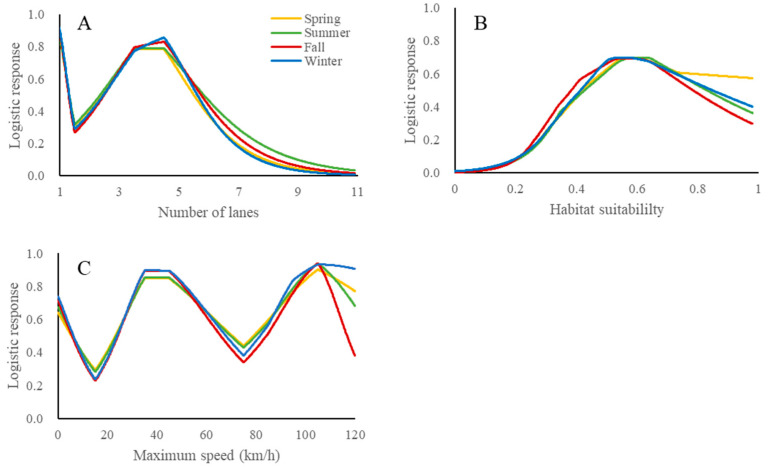
Response curves of number of lanes (**A**), habitat suitability (**B**) and maximum speed (**C**) in each season for *H. inermis* using UVC data between 2019 and 2021 in the Republic of Korea. Each response curve of predicted probability is independent of other variables in the models.

Unlike the UVC probability of *C. pygargus*, a high UVC probability of *H. inermis* was largely distributed outside the high mountain chain throughout the country (Figure 5). Furthermore, there were few changes in spatial distribution of UVC probabilities throughout the seasons. Thus, both habitat- and traffic-related variables were important for the UVC models of *H. inermis* across the seasons (Figure 6 and Table 5). For *H. inermis* UVC predictive models, the variables with the highest permutation importance were habitat suitability, maximum speed, and number of lanes for all seasons. For the traffic-related variables, UVC probabilities were high on roads with three to five lanes and with maximum speeds between 40 and 50 km/h or higher than 100 km/h.

**Figure 7:**
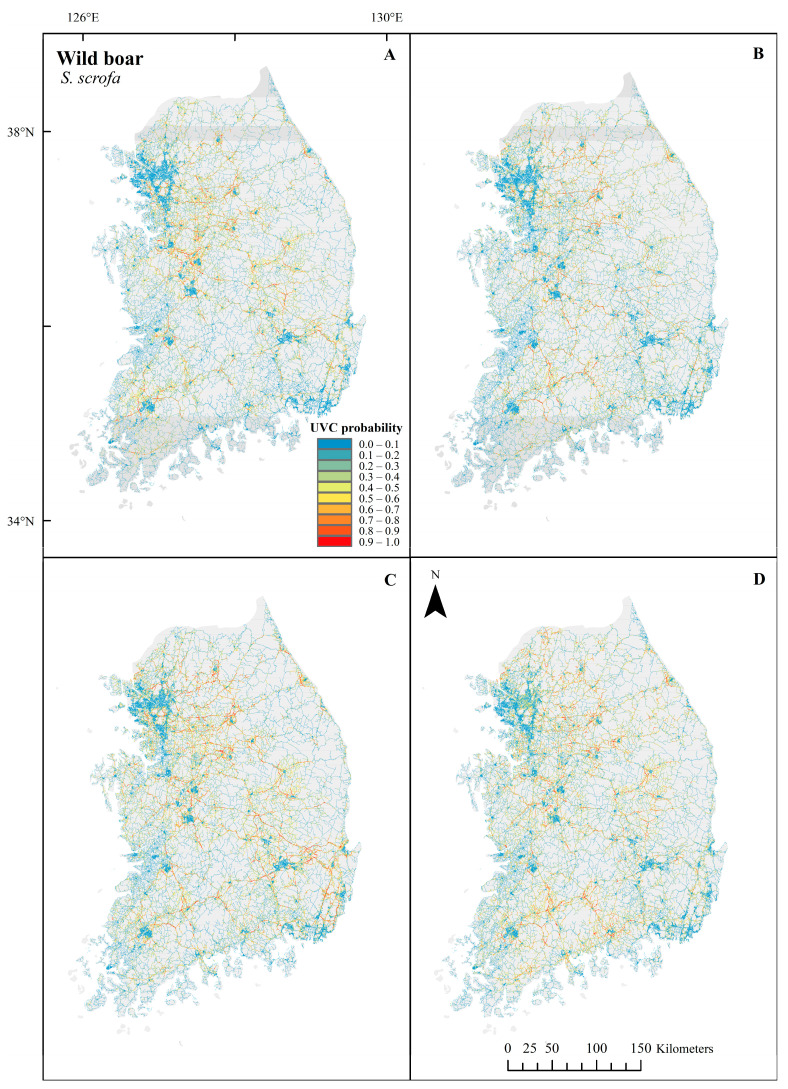
Season-based UVC probability predictions of *S. scrofa* in spring (**A**), summer (**B**), fall (**C**) and winter (**D**) along roads using UVC data between 2019 and 2021 in the Republic of Korea. Red and blue colors indicate a high and a low probability of UVC, respectively. The maps were generated using ArcGIS Desktop 10.8.1.

**Figure 8:**
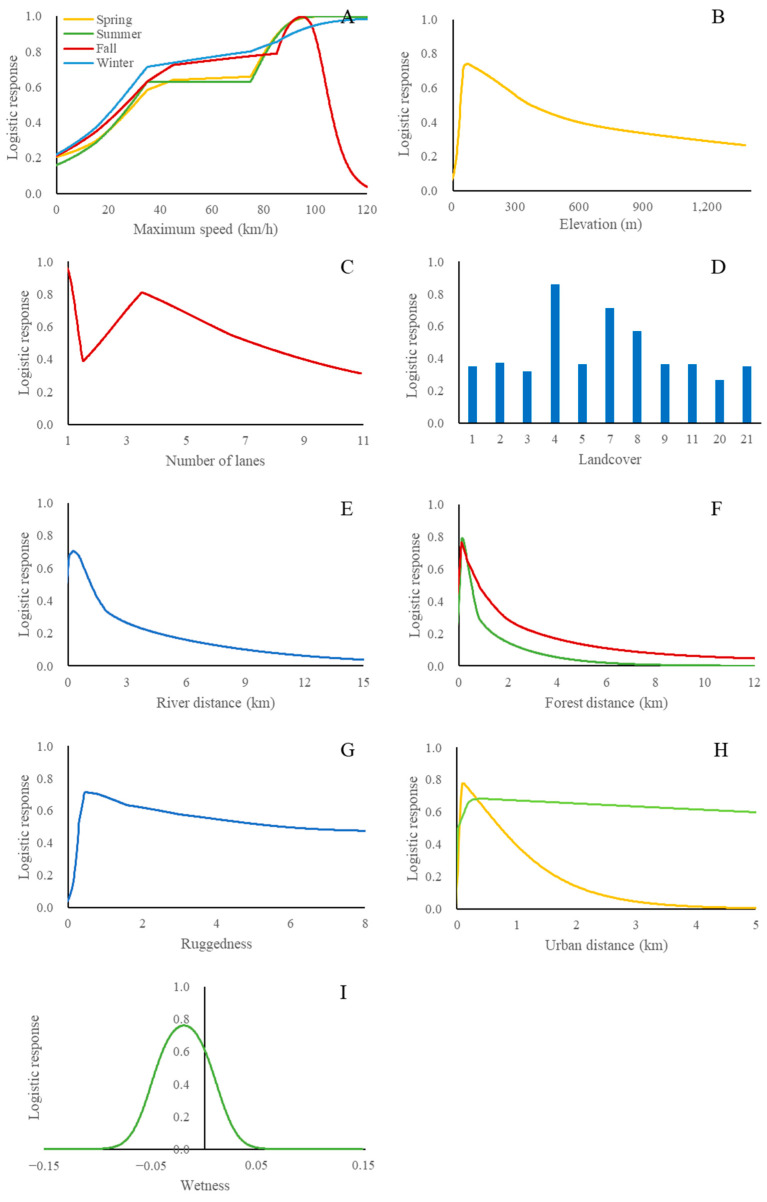
Response curves of maximum speed (**A**), elevation (**B**), number of lanes (**C**), landcover (**D**), river distance (**E**), forest distance (**F**), ruggedness (**G**), urban distance (**H**) and wetness (**I**) in each season for *S. scrofa* using UVC data between 2019 and 2021 in the Republic of Korea. Each response curve of predicted probability is independent of other variables in the models.

The UVC probabilities of *S. scrofa* fluctuated between seasons (Figure 7), and the important variables for predicting the UVC of *S. scrofa* varied by season (Figure 8 and Table 6). The variables with the highest permutation importance were elevation, maximum speed, and urban distance for spring. In summer, the variables with the highest permutation importance were wetness, maximum speed, forest distance, and urban distance. The variables with the highest permutation importance were maximum speed, number of lanes, and forest distance for fall. The variables with the highest permutation importance were river distance, maximum speed, ruggedness, and land cover for winter. Notably, although habitat suitability was not included in the modeling due to high correlations with other variables, variables related to habitat were highly influential in *S. scrofa* UVC models across seasons. Moreover, maximum speed was critical for the UVC models across all seasons. As the maximum speed increased, the UVC probabilities generally increased with a peak of approximately 90 km/h. The UVC probabilities were highest close to the forest and the river and then tended to decrease as the distance from the forest and the river increased. UVC probabilities peaked in areas with low ruggedness and a few hundred meters from urban areas in summer and then slightly decreased as the ruggedness and distance from urban areas increased. However, UVC probabilities peaked very close to urban areas and then dropped rapidly as the distance to urban areas increased in spring. UVC probabilities were generally higher in areas with low wetness. To summarize, important variables predicting UVC of *S. scrofa* fluctuated greatly by season as well as the spatial distribution of the high probability of UVC in the prediction map.

## 4. Discussion

Our results confirmed that active-season behavior was correlated with peak UVC seasons in three ungulate species (*C. pygargus*, *H. inermis*, and *S. scrofa*) in the Republic of Korea. The UVC frequencies peaked in rutting season of *C. pygargus* and *S. scrofa* and peaked in dispersal season of *H. inermis*. During the rutting and dispersal seasons, animals increase their activity and movements. In addition, dispersal of juveniles may lead to individuals that have not experienced the dangers of roads to the roads, resulting in a high risk of UVC. Thus, the UVC peak seasons coincided with the most active season for all three species, which is in line with other studies [24,50,51]. This pattern is applicable to other species. For example, in the UK, the number of European badger *Meles meles* AVC increased in spring and fall, which coincides with high-activity seasons such as mate-searching and dispersal of juveniles [24]. Therefore, we conclude that high-activity seasons are related to high UVC rates, and seasonal behaviors of animals need to be considered as one of the critical factors that may impact the temporal patterns of UVC.

The variation in the predictive power of the habitat suitability model using AUC seemed to be related to the nature of habitat use in the three ungulate species. Habitat suitability models are generally a better fit for habitat specialists, which require in a narrow range of environmental conditions, and a poorer fit for habitat generalists, which thrive in a wide variety of environmental conditions [52,53]. Although some are also found in human residential areas for *S*. *scrofa* due to their large population size in the Republic of Korea [35], *C. pygargus* and *S. scrofa* are often inhabiting internal spaces in forests. In contrast, *H. inermis* is a habitat generalist that exploits wetlands and adjacent areas even in modified habitats. Generalist species utilize various types of habitats without distinctive preferences, often resulting in poor model fits in the HSMs [54,55]. Thus, low values of model fits reflect the generalist habitat use of *H. inermis*, not negating the validity of models.

We highlight that the UVC predictive models for each species had distinctive patterns. The variables important for predicting *C*. *pygagus* UVC across seasons were related to habitat characteristics only. In contrast, the UVC models for *H*. *inermis* and *S*. *scrofa* included habitat-related and traffic-related variables. Notably, the UVC probabilities of these two species were highest for roads with a maximum speed of 100 km/h. The maximum road speeds were critical for the UVC probability models of *H. inermis* and *S. scrofa* in all seasons. For both species, UVC probabilities increased drastically at maximum speeds of 70–80 km/h or around 90–100 km/h. Vehicle speed is a critical factor that affects UVC by reducing the response time of drivers to the emergence of an animal in front of the vehicle [56,57,58]. Danks and Poter (2010) discovered that UVC probability increased drastically with an increase in speed, especially from 72 km/h onward. Thus, we assume that decreasing the maximum speed of vehicles in the road sections where UVC probability is high may contribute to UVC mitigation.

UVC probabilities were largely influenced by landscape variables in *C. pygargus*, which prefers high-elevation habitats above 200 m [59], as demonstrated in this study by habitat suitability modeling with elevation as an important parameter. A river is formed typically in the lowlands, away from the preferred habitats of *C. pygargus*. Thus, UVC probabilities of *C. pygargus* linearly increased as distance to river increased, reflecting the habitat preference of the species. UVC probability increased with an increase in habitat suitability for *C. pygargus*. High habitat suitability indicates high-quality habitat and food availability to wildlife. The number of AVC and population size were considered to be correlated [60], which could result in a high probability of UVC. From this perspective, *C. pygargus* is highly dependent on its habitat, which coincides with the ecological characteristic of this species as habitat specialist. The UVC probability also increased at low and high ruggedness and decreased at intermediate ruggedness in the fall. However, the effect of ruggedness on the UVC probability of *C. pygargus* could not be determined and further studies are necessary.

UVC predictive seasonal models of *H. inermis* included high permutation importance of the number of lanes, maximum speed, and habitat suitability for all seasons and showed similar patterns across seasons. The UVC probability of *H. inermis* was highest on four-lane roads and decreased after an increase in the number of lanes. This may be because the KROS data have been collected mainly from expressways and national highways, which are major road types in the Republic of Korea [61]. In these road types, according to the Korean Statistical Information Service, the main number of lanes is four (58.9%), followed by two (29.3%), six (7.9%), eight (3.4%), and more than ten (0.4%) [62]. For maximum speed, the predicted probability of UVC was highest at 40–50 km/h and around 100 km/h but tended to decrease at a maximum speed of less than 40 km/h, higher than 100 km/h, or between 50 km/h and 100 km/h. Roads with maximum speed limits of less than 50 km/h are usually located in the countryside. Roads in the countryside, except expressways and highways, are level with the surrounding areas, making them easily accessible to *H. inermis*. As a habitat generalist, *H. imermis* often crosses roads near or in agricultural lands, which is a preferred habitat of the species [26,63]. In addition, the UVC probability of *H. inermis* increased with an increase in habitat suitability until intermediate suitability and decreased toward high suitability. This pattern could be related to ecological traits of the species. As a habitat generalist and an edge species [26], *H*. *inermis* may not rely on habitat suitability and also inhabit near roads, where habitat is highly fragmented and habitat suitability is not high, which can lead to this result.

Compared with the other two species, the UVC seasonal models of *S. scrofa* were more complex. Except for the maximum speed, the UVC models had different variables that were important by season. *Sus scrofa* is highly flexible in habitat use [64]. This species is highly affected by the environment, food availability, seasonal changes, and anthropogenic disturbances [65,66,67]. These characteristics make this species susceptible to environmental changes. For instance, although *S*. *scrofa* typically inhabits the forest interior, the UVC probability peaked around grasslands and agricultural lands in winter, when food is scarce. Additionally, the predicted probability of UVC was highest at approximately 90–100 km/h. Another study also concluded that vehicle speed is an important factor in predicting the UVC probability of *S. scrofa*, and an increase in speed increases UVC probability [68]. Moreover, such a high speed limit implies an expressway in the Republic of Korea, and the UVC frequency of *S. scrofa* on the expressway significantly increased between 2004 and 2019 [69]. Therefore, we suggest using the maximum speed limit for regulating the UVC occurrences involving *S. scrofa*, along with increasing driver’s awareness by using warning signs.

## 5. Conclusions

The three target species in this study had the largest body size among all vertebrates inhabiting Republic of Korea, which means that human safety is also critically threatened when a collision occurs. Therefore, it is necessary to understand the spatio-temporal characteristics of each species in order to maintain human safety on roads and for wildlife conservation. In this study, UVC was predicted using various factors related to traffic, species habitat, and spatial distribution with high probability of UVC fluctuation according to the seasonal behavior of the focal species. To reduce such threats to humans and to conserve wildlife, it is important to consider the environmental features surrounding roads and traffic factors. Based on our results, we suggest the following methods to mitigate UVC of the three species: For *C*. *pygargus*, fencing along roads where UVC probability is highly predicted would reduce UVC of the species. For *H*. *inermis*, on small roads, fencing along roads where speed limit is between 40 and 50 km/h and, in large roads, lowering speed limits where the speed limit is above 100 km/h, especially in four-lanes roads, will be helpful for mitigating UVC of this species. For *S*. *scrofa*, lowering speed limits in areas where UVC probability is highly predicted would contribute to reducing UVC in all seasons. Utilizing and applying the distinctive variables important to predict UVC across seasons for each species will be helpful in planning road safety and conservation actions.

## Figures and Tables

**Table 1:** Variables used for modeling habitat suitability and UVC probability of three ungulate species between 2019 and 2021 in the Republic of Korea.

Category	Variable	Model Used	Explanation
Bioclimate	Annual mean temperature	HSM, UVC	-
Bioclimate	Mean diurnal range	HSM	Mean of monthly (max temp −min temp)
Bioclimate	Isothermality	HSM	Mean diurnal range/Temperature annual range ×100
Bioclimate	Temperature seasonality	HSM	Standard deviation × 100
Bioclimate	Maximum temperature of warmest month	HSM	-
Bioclimate	Minimum temperature of coldest month	HSM	-
Bioclimate	Temperature annual range	HSM	Maximum temperature of warmest month- Minimum temperature of coldest month
Bioclimate	Mean temperature of wettest quarter	HSM	-
Bioclimate	Mean temperature of driest quarter	HSM	-
Bioclimate	Mean temperature of warmest quarter	HSM	-
Bioclimate	Mean temperature of coldest quarter	HSM	-
Bioclimate	Annual precipitation	HSM, UVC	-
Bioclimate	Precipitation of wettest month	HSM	-
Bioclimate	Precipitation of driest month	HSM	-
Bioclimate	Precipitation seasonality	HSM	Coefficient of variation
Bioclimate	Precipitation of wettest quarter	HSM	-
Bioclimate	Precipitation of driest quarter	HSM	-
Bioclimate	Precipitation of warmest quarter	HSM	-
Bioclimate	Precipitation of coldest quarter	HSM	-
Habitat	Elevation	HSM, UVC	Digital Elevation Model (DEM; GMTED provided by USGS) smoothed using focal statistics
Habitat	Slope	HSM, UVC	Derived from DEM using Slope tool in ArcMap
Habitat	Ruggedness	HSM, UVC	Derived from DEM—standard deviation of slope using focal statistics
Habitat	Habitat landcover	HSM, UVC	Extracted from World Land Cover 30 m BaseVue 2013, Source: MDAUS
Habitat	Forest distance	HSM, UVC	Euclidean distance to forest land cover class (BaseVue 2013)
Habitat	Urban distance	HSM, UVC	Euclidean distance to urban land cover class (BaseVue 2013)
Habitat	Water distance	HSM, UVC	Euclidean distance to water cover class (BaseVue 2013)
Habitat	River distance	HSM, UVC	Euclidean distance to streams/rivers
Habitat	River-coast distance	HSM, UVC	Euclidean distance to streams/rivers or coast
Habitat	Greenness	HSM, UVC	Vegetation index, derived using Tasselled Cap transformation of corrected Landsat reflectance imagery
Habitat	Wetness	HSM, UVC	Surface and canopy moisture, derived using Tasselled Cap transformation of corrected Landsat reflectance imagery
Habitat	Corridor distance	UVC	Euclidean distance to wildlife crossings
Traffic	Lanes	UVC	Number of lanes per road (two-way)
Traffic	Max speed	UVC	Maximum road speed

**Table 2:** Land cover categories included in BaseVue (2013).

Category	Description
1	Deciduous forest
2	Evergreen forest
3	Shrub/Scrub
4	Grassland
5	Barren or minimal vegetation
7	Agriculture, general (cultivated crop lands)
8	Agriculture, paddy (crop lands characterized by inundation)
9	Wetland
11	Water
20	High density urban (over 70% constructed materials)
21	Medium-low density urban (30–70% constructed materials)

**Table 3:** Area under the receiver operating curve (AUC) and true scale statistics (TSS) of 12 UVC probability models between 2019 and 2021 in the Republic of Korea. Values (mean ± standard error) for spring, summer, fall, and winter UVC in each species.

	Spring	Summer	Fall	Winter
*C. pygargus*				
AUC	0.877 ± 0.032	0.910 ± 0.026	0.923 ± 0.032	0.962 ± 0.020
TSS	0.597 ± 0.091	0.625 ± 0.038	0.804 ± 0.102	0.845 ± 0.072
*H. inermis*				
AUC	0.750 ± 0.007	0.752 ± 0.007	0.785 ± 0.010	0.791 ± 0.010
TSS	0.518 ± 0.020	0.515 ± 0.018	0.516 ± 0.013	0.506 ± 0.012
*S. scrofa*				
AUC	0.785 ± 0.065	0.817 ± 0.055	0.794 ± 0.035	0.754 ± 0.067
TSS	0.434 ± 0.180	0.444 ± 0.241	0.436 ± 0.114	0.320 ± 0.090

**Table 4:** Permutation importance (%) of each variable included in the seasonal models of *C. pygargus* generated using UVC data between 2019 and 2021 in the Republic of Korea. The highest values (>10) for each model are presented in bold.

Parameter	Spring	Summer	Fall	Winter
Annual mean temperature	3.4	4.3	0.9	1.5
Annual precipitation	1.2	0.4	0.3	1.1
Corridor distance	0.9	0.8	2.1	0.5
Elevation	2.2	1.5	0.9	0.9
Forest distance	6.3	2.5	0.4	0.4
Greenness	5.7	3.3	1.5	0.4
Landcover	2.1	2.8	1.6	0.1
River distance	**18.3**	**17.9**	**29.4**	**82.9**
River-coast distance	1.8	0.2	1.5	0.2
Habitat suitability	**42.1**	**58.4**	**39.0**	8.2
Ruggedness	5.2	1.0	**10.5**	0.8
Slope	2.1	1.2	3.2	0.3
Urban distance	1.3	1.6	1.2	0.6
Water distance	1.2	0.3	1.7	0.1
Wetness	0.8	1.1	0.9	0.4
Maximum speed	2.5	2.1	2.8	0.6
Lanes	3.0	0.6	2.1	1.2

**Table 5:** Permutation importance (%) of each variable included in the seasonal models of *H. inermis* generated using UVC data between 2019 and 2021 in the Republic of Korea. The highest values (>10) for each model are presented in bold.

Parameter	Spring	Summer	Fall	Winter
Annual mean temperature	8.8	7.1	5.5	8.2
Annual precipitation	1.2	1.3	1.1	3.7
Elevation	2.2	1.7	1.8	3.7
Forest distance	0.9	0.9	0.6	0.6
Greenness	2.8	2.3	5.7	3.1
Landcover	5.7	5.2	8.9	8.7
River distance	0.3	0.6	1.5	0.9
River-coast distance	0.7	1.4	0.5	1.1
Habitat suitability	**30.5**	**32.9**	**25.0**	**23.2**
Ruggedness	0.4	0.6	0.7	0.1
Slope	0.4	0.4	0.2	1.1
Urban distance	0.8	1.1	0.9	2.3
Water distance	0.9	0.4	0.4	0.4
Wetness	1.3	1.3	2.1	2.6
Corridor distance	0.4	0.6	0.8	1.3
Maximum speed	**24.3**	**26.7**	**27.4**	**22.8**
Lanes	**18.3**	**15.5**	**16.8**	**16.3**

**Table 6:** Permutation importance (%) of each variable included in the seasonal models of *S. scrofa* generated using UVC data between 2019 and 2021 in the Republic of Korea. Habitat suitability is excluded due to high correlations with other variables. The highest values (>10) for each model are presented in bold.

Parameter	Spring	Summer	Fall	Winter
Annual mean temperature	1.2	0.7	0.4	0.3
Annual precipitation	1.0	1.3	1.5	2.9
Corridor distance	0.2	0.2	3.9	1.3
Elevation	**28.7**	4.0	5.7	4.8
Forest distance	8.7	**13.1**	**12.7**	7.4
Greenness	0.7	0.3	4.2	0.3
Landcover	1.6	5.7	7.7	**11.2**
River distance	9.7	7.6	4.8	**17.4**
River-coast distance	3.3	0.1	4.6	2.0
Ruggedness	3.5	2.1	5.2	**13.6**
Slope	3.8	1.8	1.2	1.1
Urban distance	**14.2**	**12.8**	3.8	6.0
Water distance	1.1	8.9	3.1	1.5
Wetness	4.8	**22.7**	8.4	9.4
Maximum speed	**14.8**	**16.9**	**18.1**	**14.8**
Lanes	2.8	1.6	**14.7**	5.9

## Data Availability

The datasets generated and/or analyzed during the current study are available from the National Institute of Ecology on reasonable request.
